# Overexpression of calcitonin gene-related peptide protects mouse cerebral microvascular endothelial cells from high-glucose-induced damage via ERK/HIF-1/VEGF signaling

**DOI:** 10.1007/s12576-019-00708-2

**Published:** 2019-09-05

**Authors:** Yanjun Guo, Qin Zhang, Huilu Chen, Yixuan Jiang, Ping Gong

**Affiliations:** 1 State Key Laboratory of Oral Diseases, National Clinical Research Center of Oral Diseases, West China Hospital of Stomatology, Sichuan University, Chengdu, China; 2 Department of Implantology, West China Hospital of Stomatology, Sichuan University, Chengdu, China

**Keywords:** Calcitonin gene-related peptide, Diabetes mellitus, Neovascularization, Reactive oxygen species

## Abstract

In the diabetic brain, hyperglycemia damages the cerebrovasculature and impairs neurovascular crosstalk. Calcitonin gene-related peptide (CGRP) is an important neuropeptide that is active in the vascular system. In this study, we aimed to investigate whether CGRP is involved in the high-glucose-induced damage in mouse cerebral microvascular endothelial (b.END3) cells and the possible mechanism in vitro. The overexpression of CGRP by lentiviral transduction inhibited cell apoptosis but not proliferation. In contrast to the promoting of angiogenesis and migration under normal glucose, CGRP inhibited hyperglycemia-induced tube formation but had no effect on migration. Calcitonin gene-related peptide partly reduced the increased level of intracellular reactive oxygen species (ROS) and altered nitric oxide synthase mRNA expression. Furthermore, CGRP suppressed the increased HIF-1α/VEGF-A expression and the phosphorylation of ERK1/2 in hyperglycemia. The ERK inhibitor U0126 showed similar inhibition of cell apoptosis, tube formation and HIF-1α/VEGF expression as that exhibited by lenti-CGRP. These findings demonstrate the protective role of CGRP overexpression against high-glucose-induced cerebrovascular changes in b.END3 cells, possibly through the inhibition of ERK/HIF-1/VEGF signaling.

## Introduction

Among the many complications of diabetes mellitus, vascular dysfunction plays a primary role and often begins with endothelial cell damage [1]. Diabetic endothelial cells exhibit a series of changes, such as reduced nitric oxide (NO) activity and increased cytokines and reactive oxygen species (ROS) levels, and oxidative stress plays a central role in these changes [2–5]. In addition, the antioxidant defense system, which includes Superoxide Dismutase (SOD), is also influenced [3].

Compared to macrovascular complications, microvascular changes are prone to respond more quickly to microenvironmental stimuli in the elaborate vascular system such as in the brain [6]. Under hyperglycemia, the brain endothelial cells exhibit decreased viability, increased apoptosis and severe oxidative damage [7–9].

The major mechanisms underlying the changes in cerebral microvascular endothelial cells under high glucose remain unclear. The ERK cascades are mainly considered to be correlated with cell proliferation and survival, inflammatory cytokines, actin remodeling, and angiogenesis and are, therefore, considered the major regulators of the oxidative response in endothelial cells [10]. Exposure to high glucose can activate the phosphorylation of ERK1/2 in vascular cells [11, 12]. Furthermore, the activation of ERK upregulates the expression of hypoxia inducible factor-1 (HIF-1) followed by vascular endothelial growth factor (VEGF), which are reported to be important regulators in diabetic cerebral microvascular endothelial cells [7, 13]. Early vascular changes, which are accompanied by the upregulation of HIF-1, suggest that the relative hypoxic microenvironment may be the initial cause of cerebral vascular dysfunction [14]. Accordingly, the increased expression of VEGF results in proliferative angiogenesis [13, 15].

Calcitonin gene-related peptide (CGRP), a 37-amino-acid neuropeptide, is widely distributed in the central and peripheral nervous systems, as well as nonneuronal cells such as endothelial cells [16]. The CGRP receptors, including receptor-activity-modifying proteins (RAMPs) and calcitonin receptor-like receptor (CRLR), have been confirmed to be present in cerebral microvascular endothelial cells in culture, and RAMPs may be the main receptors that execute biological functions [17, 18]. Calcitonin gene-related peptide has strong vasodilative activity in many vascular systems, such as the cardiovasculature and cerebrovasculature [18, 19]. Calcitonin gene-related peptide also plays a role in the angiogenesis, proliferation and migration of human umbilical vein endothelial cells (HUVECs) and appears to protect against cell death and intracellular ROS in cardiomyocytes and vascular smooth muscle cells through ERK1/2 signaling [20–23]. However, the impact of CGRP on cerebral microvascular endothelial cells under high glucose conditions has been rarely investigated. In the diabetic brain, the expression of CGRP is severely decreased but is different in the various cerebral areas [24]. Diabetes is also linked to sensory neuropathy with sensory nerve degeneration and a loss of CGRP-containing sensory neurons. Calcitonin gene-related peptide may have a role in diabetic neuropathy, as the overexpression of CGRP has been reported to protect Schwann cell lines from oxidative stress under high glucose conditions [25]. Interestingly, diabetes increases the risk of neurologic disorders in the brain, which are all related to the cerebrovasculature [14]. Hence, we are interested in determining how CGRP, as a neuropeptide and vasoactive factor, reacts to high glucose conditions in cerebral microvascular endothelial cells. In the present study, we investigated the effect of the overexpression of CGRP in mouse cerebral microvascular endothelial cells (b.END3) cells and whether it is associated with ERK/HIF-1/VEGF signaling.

## Materials and methods

### Cell culture and the construction of a lentiviral vector system

Mouse cerebral microvascular endothelial (b.END3) cells were purchased from American Type Culture Collection (ATCC, Molsheim Cedex, France) and cultured in Dulbecco’s modified Eagle’s medium (DMEM) (HyClone; Logan, Utah, USA) supplemented with 10% fetal bovine serum (FBS) (Biowest; France), and 1% 100 IU/ml penicillin/streptomycin (HyClone) at 37 °C at 5% CO_2_. The cells were passaged with 0.25% EDTA-trypsin (HyClone), and when they reached confluency, the growth medium was changed every 3 days. The ERK1/2 inhibitor used in this study was U0126 (10 µM, Cell Signaling Technology, #9903, Danvers, USA).

The CGRP overexpression lentiviral vector was constructed and administered as previously reported [26]. b.END3 cells were transfected with the lentiviral vector for 8 h at MOI = 10. The stable overexpression of CGRP was subsequently confirmed by quantitative real-time PCR and western blotting.

### Immunofluorescence staining

The b.END3 cells from different groups were seeded on 48-well plates and fixed in 4% paraformaldehyde at room temperature for 15 min. The cells were then blocked with 5% BSA/PBS for 1 h at room temperature and incubated with diluted primary antibodies Ramp1 and HIF-1α (1:200, Santa Cruz, CA, USA) at 4 °C overnight. The next day, corresponding conjugated secondary antibodies conjugated (1:500, Life Technologies, USA) were added to each well for a 1 h incubation in the dark. DAPI was then used to stain the nuclei. Ramp1 and HIF-1α were observed as red fluorescence, and the nuclei were observed as blue fluorescence under an Olympus IX71 fluorescence microscope (Olympus, Japan).

### RNA isolation and quantitative real-time PCR

Total RNA was extracted by Trizol reagent (Invitrogen, USA), and the reverse transcription was performed with the PrimeScript™ RT reagent Kit with gDNA Eraser (Perfect Real Time) (TaKaRa, Japan) according to the manufacturer’s protocol. RT-qPCR was carried out on an ABI 7900 system with TB Green™ Premix Ex Taq™ II (Tli RNaseH Plus) (TaKaRa, Japan). The relative mRNA level was normalized to GAPDH as the reference gene. The relative changes in mRNA expression were analyzed using the 2^−∆∆Ct^ method. The primer sequences are shown in Table 1.

**Table 1:** Sequences of the primers used for qPCR

Gene	Forward (5′–3′)	Reverse (5′–3′)
Ramp1	CACGGTGTGACCTGGTATTG	TTTGAAAGGAGACCCATTGC
HIF-1α	TCAGCATACAGTGGCACTCA	AAGGGAGCCATCATGTTCCA
VEGF	ACTGGACCCTGGCTTTACTGCT	TGATCCGCATGATCTGCATGGTG
VEGFR1	GTGTCTATAGGTGCCGAGCC	CGGAAGAAGACCGCTTCAGT
VEGFR2	GGAGAAGAATGTGGTTAAGATCTGTGA	ACACATCGCTCTGAATTGTGTATACT
iNOS	TCCCAAGTACGAGTGGTTCC	TGGTCACATTCTGCTTCTGG
eNOS	GGGTGTTTGGACAAGTCCTC	AGTCCGAAAATGTCCTCGTG
GAPDH	AAGGCCGGGGCCCACTTGAA	GGACTGTGGTCATGAGCCCTTCCA

### Western blotting

Cells were scraped in lysis buffer and centrifuged at 12,000×*g* at 4 °C for 10 min. The protein concentration was measured by the bicinchoninic acid (BCA) method (Beyotime, Shanghai, China). Proteins were separated by SDS-PAGE, electrophoresed, blotted onto a PVDF membrane, blocked with 5% BSA/TBST for 1 h and then incubated with primary antibody at 4 °C overnight. The next day, the membrane was washed with TBST for three times and then incubated with secondary antibody (1:5000 dilution) (SAB, Maryland, USA) for 1 h. The membrane was again washed with TBST for three times. The bands were visualized using an enhanced chemiluminescence kit (Beyotime, Shanghai, China) and imaged with QuantityOne software (Bio-Rad, California, USA). The primary antibodies used in this study were as follows: CGRP (1:200, Santa Cruz, #sc-28920), SOD2 (1:500, Santa Cruz, #sc-133134), HIF-1α (1:400, Novus Biologicals, #NB100-105), p-ERK1/2 (1:500, Santa Cruz, #sc-81492), ERK1/2 (1:500, Santa Cruz, #sc-514302), p-p65 (1:1000, Cell Signaling Technology, #3033), p65 (1:1000, Cell Signaling Technology, #8242), and GAPDH (1:1000, SAB, #40493).

### Cell counting kit-8 (CCK-8)

The CCK-8 method was performed to observe the cell viability under different treatments. Cells were plated at a density of 5000 cells per well. After adherence, the cells were starved for 24 h. On days 1, 3, and 5, 10 µl of CCK-8 reagent (Dojindo; Japan) and 90 µl of serum-free medium was added to each well and the cells were incubated for 40 min at 37 °C. The optical density (OD) value was determined by a microplate reader at 490 nm.

### Annexin V-FITC/PI cell apoptosis assay

We used annexin V-FITC/PI double staining to determine cell apoptosis by flow cytometry according to the manufacturer’s instructions (SAB, Maryland, USA). Briefly, after incubation with 25 mM or 50 mM glucose with 5% serum for 5 days, the cells were digested by trypsin without EDTA (Solarbio, Beijing, China), collected and resuspended in 1 × binding buffer. Then, annexin V was added to the binding buffer and incubated in the dark for 15 min at 4 °C. PI was then added and incubated for another 5 min. The samples were then analyzed by flow cytometry within 30 min.

### Tube formation assay

The cells used in this assay were pretreated with 25 mM or 50 mM glucose for 3 days. On ice, 96-well plates were coated with 50 µl of Growth Factor Reduced Matrigel™ Matrix (#356230, BD Bioscience; USA) per well. A total of 40,000 cells were seeded in each well and incubated under 25 mM or 50 mM glucose at 37 °C in 5% CO_2_ for 6 h. Tube formation was observed under phase-contrast microscopy, and the number of nodes and the length of the formed tubes were calculated with Image-Pro Plus 6.0 software (Media Cybernetics; Rockville, USA).

### Cell migration

The cells used in this assay were pretreated with 25 mM or 50 mM glucose for 3 days. Fifty thousand b.END3 cells suspended in 1% FBS were placed in the upper transwell chamber and growth medium containing 10% FBS was placed in the lower chamber. After incubation for 24 h, the cells on the upper surface were removed, and the chamber was fixed with 4% paraformaldehyde for 10 min and stained with crystal violet for 30 min. The mean number of cells in five random fields was counted under a confocal microscope (200 ×).

### Detection of intracellular ROS

The intracellular ROS level was determined by the fluorescent probe dihydroethidium (DHE) (Beyotime, Shanghai, China). The cells were incubated with 10 µM of DHE in serum-free medium at 37 °C for 30 min. After washing with serum-free medium for three times, the fluorescent intensity of ROS was observed under a confocal microscope. The images were taken with the same exposure settings for each group.

### Enzyme-linked immunosorbent assay (ELISA)

After incubation with 25 mM or 50 mM glucose for 3 days, the cell culture supernatants from the different groups were collected for BCA protein quantitative analysis (Beyotime, Shanghai, China). For each group, 100 µl of supernatant was used for the detection of secreted VEGF by an ELISA kit (SAB, Maryland, USA) according to the manufacturer’s instructions.

### Statistical analysis

Statistical analysis was carried out with SPSS 21.0 (SPSS Inc., Chicago, IL). The results are expressed as the mean ± SEM. Statistical analysis was performed using unpaired *t* test, one-way analysis of variation (ANOVA) or two-way ANOVA when appropriate followed by the Student–Newman–Keuls test for multiple comparisons. Differences with *p* < 0.05 were considered statistically significant.

## Results

### High glucose decreased the expression of the main CGRP receptor, and successful lentiviral transduction led to the stable overexpression of CGRP in b.END3 cells

**Fig. 1:**
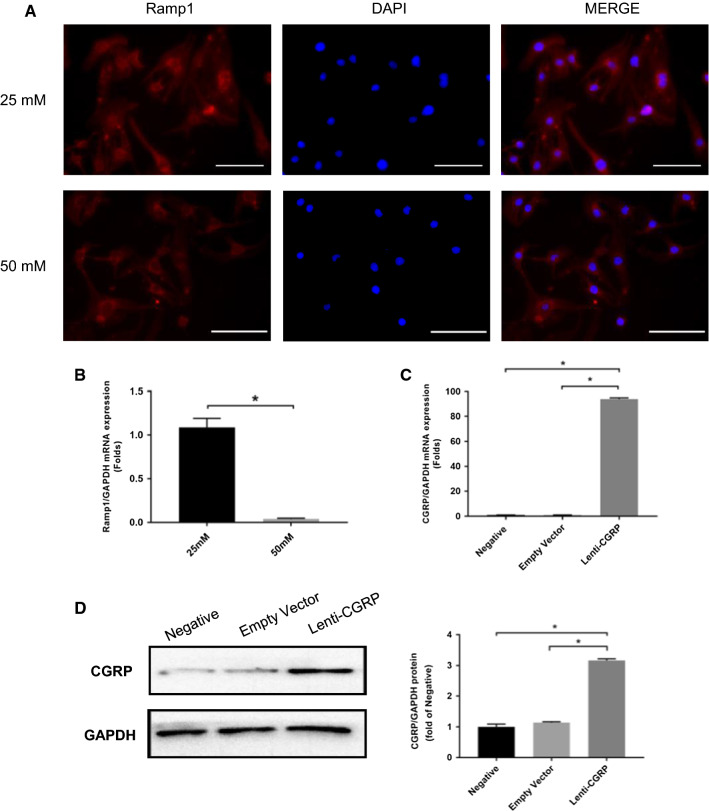
High glucose exposure decreased the expression of the main CGRP receptor, and the successful lentiviral transduction led to the stable overexpression of CGRP in b.END3 cells. **a** Immunofluorescence staining of the main CGRP receptor Ramp1 under high glucose for 3 days. Scale bar = 50 μm. **b** The mRNA expression of Ramp1 under 25 mM and 50 mM glucose conditions after high glucose treatment for 3 days. **c** The mRNA expression of the negative control group, empty vector-transfected group and lenti-CGRP-transfected group. The negative control group refers to the group that did not undergo any treatment or transduction. **d** Representative western blotting images of CGRP protein expression in the negative control group, empty vector-transfected group and lenti-CGRP-transfected group. GAPDH was used as a reference protein control on the same membrane. All data are shown as the mean ± SEM, *p < 0.05

As shown in Fig. 1a, b, the immunofluorescence staining and the mRNA expression of the main CGRP receptor Ramp1 indicated that the 3 day high glucose incubation decreased the expression of CGRP receptors in b.END3 cells to some extent. Considering the loss of CGRP under high glucose conditions, we transfected the b.END3 cells with empty vector and the CGRP overexpression lentivirus (lenti-CGRP). The negative group represents the cells that did not undergo lentiviral transduction. The efficiency of transduction was confirmed by qPCR, and the protein expression of CGRP was determined by western blot. The results indicated that, compared with the empty vector, lenti-CGRP specifically increased the mRNA expression (Fig. 1c) and protein expression of CGRP (*p *< *0.05*) (Fig. 1d). The results indicated the overexpression of CGRP in b.END3 cells and the successfully transfected cells were used in all the subsequent experiments.

### Lenti-CGRP had no effect on cell viability but displayed an anti-apoptotic effect in high glucose

**Fig. 2:**
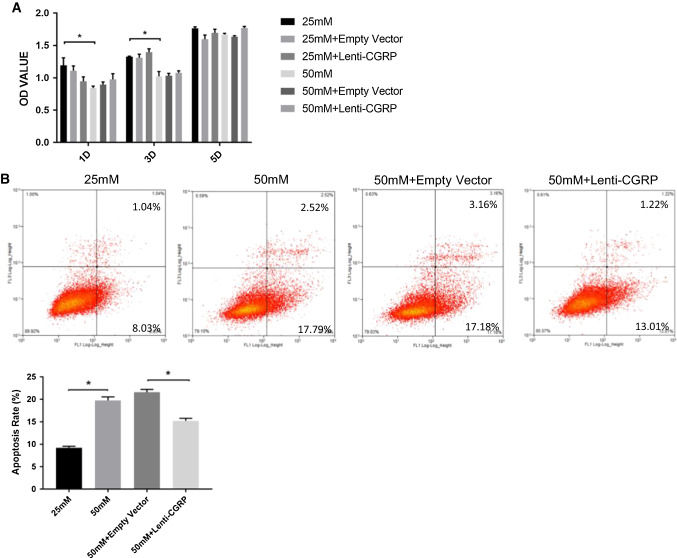
Lenti-CGRP had no effect on cell viability but displayed an anti-apoptotic effect under high glucose conditions. **a** The results of the CCK-8 assay are expressed as OD values. b.END3 cells from different groups were treated with 25 mM glucose and 50 mM glucose for 1, 3, and 5 days. The data are shown as the mean ± SEM, *p < 0.05. **b** Representative flow cytometry images of cell apoptosis, as measured by the annexin V/PI method. Cells from different groups were treated with 25 mM or 50 mM glucose for 5 days. The graph shows the apoptosis rates of the different groups. The data are shown as the mean ± SEM, *p < 0.05

The CCK-8 assay was used to compare cell viability at different time points (1, 3, and 5 days) under different glucose concentrations (Fig. 2a). Treatment with high glucose (50 mM) impaired cell viability from day 1 to day 3 (*p *< 0.05) and the difference between the 25 mM group and the 50 mM group was smaller on day 5 (*p *> 0.05). Lenti-CGRP showed no effect on cell viability under normal glucose or high glucose conditions. In addition, we confirmed that the transduction of the lentivirus did not influence cell growth.

In this study, the annexin V-FITC/PI method was used to determine the cell apoptosis by flow cytometric analysis. As shown in Fig. 2b, the early cell apoptosis rate and the late cell apoptosis in the high glucose group were nearly twofold greater than that in the normal glucose group (*p *< 0.05). Although lentivirus transduction led to a moderate increase in the apoptosis rate (*p *> 0.05), the lenti-CGRP group still displayed a greater anti-apoptotic effect than that in the empty vector group (*p *< 0.05).

### Lenti-CGRP ameliorated the proliferated tube formation but had no effect on cell migration under high glucose conditions

**Fig. 3:**
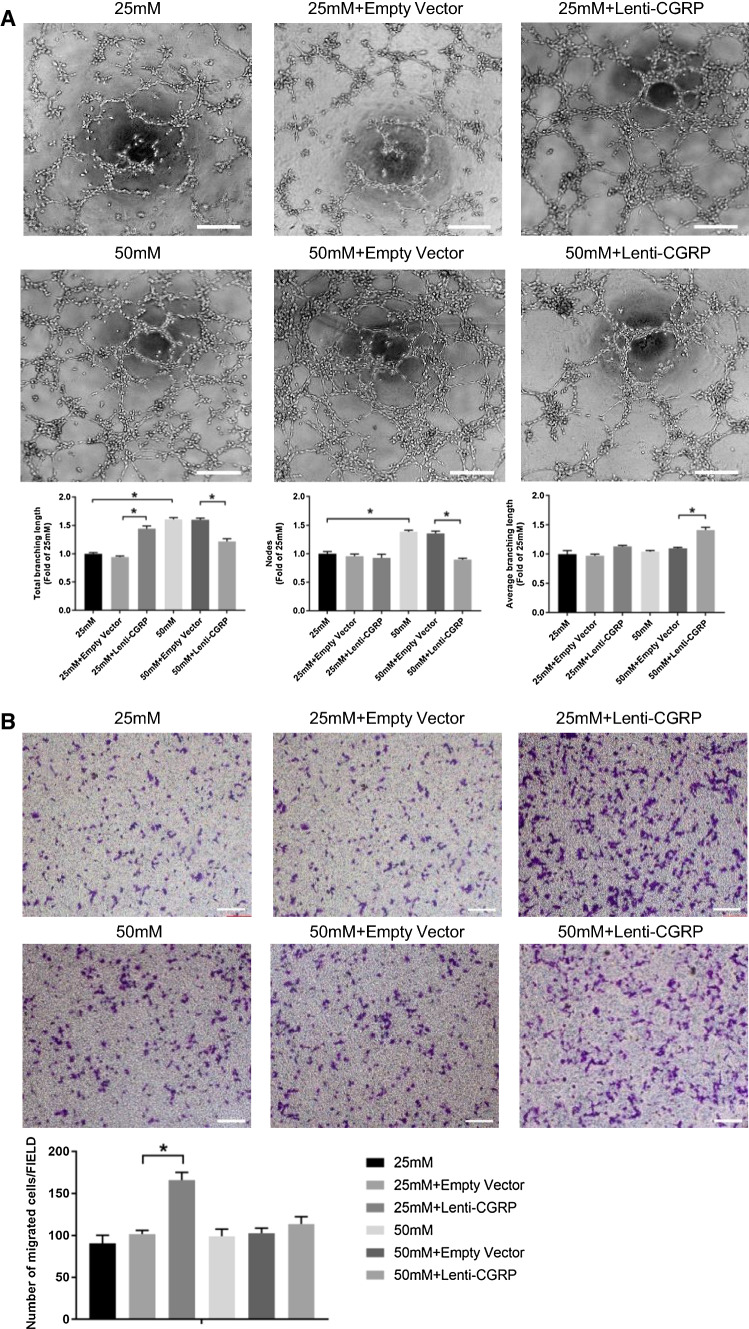
Lenti-CGRP ameliorated the proliferative tube formation but had no effect on cell migration under high glucose conditions. **a** Representative images of the Matrigel assay. The cells were pretreated with 25 mM or 50 mM glucose for 3 days. The data are shown as the fold changes compared to the 25 mM-treated control group and are expressed as the mean ± SEM, *p < 0.05. Scale bar = 200 μm. **b** Typical images of the transwell assay after 24 h glucose treatment. The cells were pretreated with 25 mM or 50 mM glucose for 3 days. The migrated cells were stained by crystal violet. Migrated cells in five random fields from each group were counted in Image-Pro Plus 6.0 by under 200 × magnification. Scale bar = 200 μm. The data are shown as the mean ± SEM, *p < 0.05

The results of the Matrigel assay are shown in Fig. 3a. Incubation with high glucose (50 mM glucose group) stimulated increased vessel formation in nodes and total branching length (*p *< 0.05) compared to that stimulated by incubation with normal glucose (25 mM group). Under normal glucose conditions, lenti-CGRP enhanced tube formation by increasing the total branching length (*p *< 0.05). Conversely, under high glucose conditions, lenti-CGRP inhibited node formation and reduced the total branching length (*p *< 0.05), while lenti-CGRP extended the average branching length (*p *< 0.05).

Cell migration was tested by the transwell assay, as shown in Fig. 3b. Under normal glucose conditions (25 mM), lenti-CGRP significantly enhanced cell migration (*p *< 0.05). Compared to treatment with normal glucose, treatment with high glucose moderately increased the number of migrated cells (*p *> 0.05), and we did not observe an obvious enhancement of migration by lenti-CGRP under high glucose exposure (*p *> 0.05).

### The protective role of lenti-CGRP against high-glucose-triggered ROS and oxidation-related factors

**Fig. 4:**
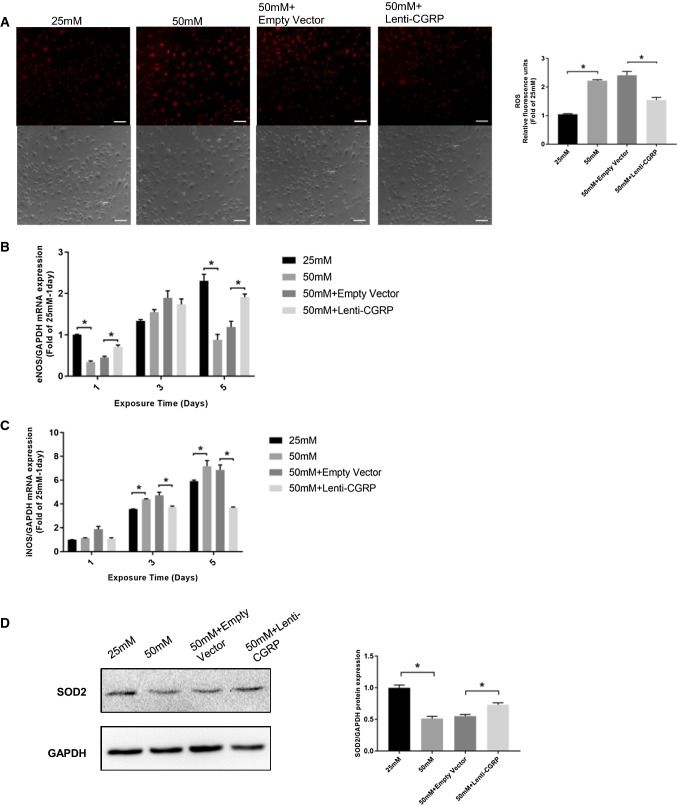
The protective role of lenti-CGRP on high-glucose-triggered ROS and oxidation-related factors. **a** Representative fluorescence images of intracellular ROS. The upper images are fluorescence images and the lower images are bright field images as controls. The relative fluorescence values were measured by Image-Pro Plus 6.0 software and are expressed as the fold change compared to the 25 mM-treated control group. The data are shown as the mean ± SEM, *p < 0.05. Scale bar = 50 μm. **b**, **c** The mRNA expression of eNOS and iNOS under high glucose conditions on days 1, 3, and 5. The data are shown as the mean ± SEM, *p < 0.05. **d** A western blotting image of MnSOD (SOD2) expression. The data are shown as the mean ± SEM, *p < 0.05

The level of intracellular ROS in b.END3 cells was determined by the fluorescent probe DHE and was observed under fluorescence microscopy. The intracellular ROS level in the 50 mM glucose-treated group was significantly increased by approximately twofold the level in the 25 mM glucose-treated control group and was partly blocked by lenti-CGRP (*p *< 0.05) (Fig. 4a). Through qPCR analysis, the mRNA expression of inducible nitric oxide synthase (iNOS) was found to be increased by 50 mM glucose on day 3 and day 5 (*p *< 0.05), while the mRNA expression of endothelial nitric oxide synthase (eNOS) was decreased under high glucose conditions mainly on day 1 and day 5 (*p *< 0.05). Lenti-CGRP significantly reduced iNOS mRNA expression from day 3, and the effect increased with time (*p *< 0.05). However, we did not observe an obvious effect of lenti-CGRP on the first day of high glucose exposure (*p *> 0.05). Conversely, lenti-CGRP upregulated the decreased eNOS mRNA expression on day 1 and day 5 (*p *< 0.05) (Fig. 4b, c). In addition, the protein expression of MnSOD (SOD2) was also detected by western blotting. The results showed that lenti-CGRP could rescue the high-glucose-induced reduction in MnSOD expression at the protein level (*p *< 0.05) (Fig. 4d).

### Lenti-CGRP attenuated HIF-1α/VEGF expression induced by high glucose

**Fig. 5:**
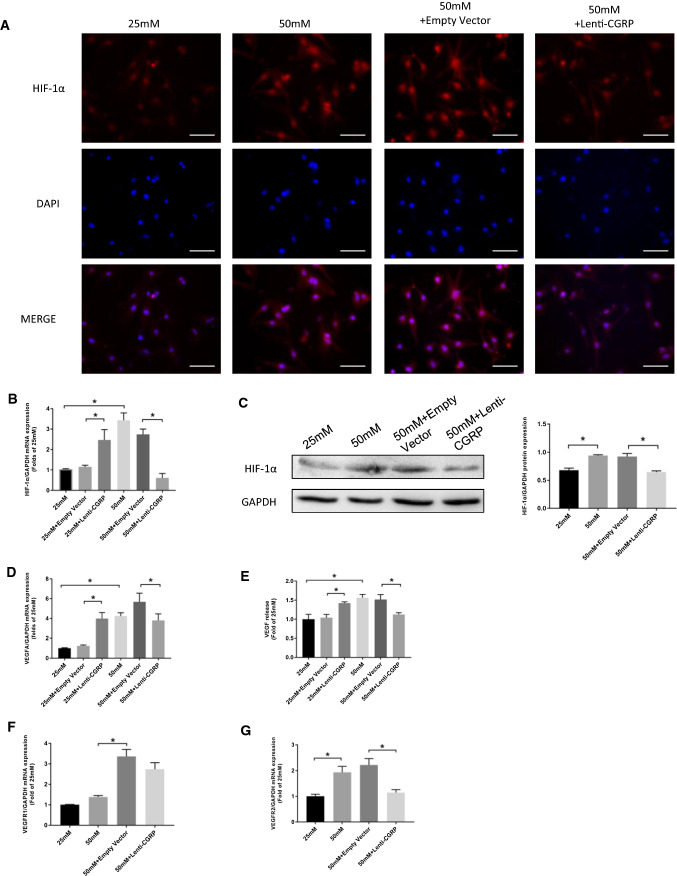
CGRP mediated HIF-1α/VEGF expression in b.END3 cells exposed to high glucose. **a** Immunofluorescence staining of HIF-1α. Scale bar = 50 μm. **b** The HIF-1α mRNA expression under high glucose conditions for 3 days. The data are shown as the fold change compared to the 25 mM-treated control group and are expressed as the mean ± SEM, *p < 0.05. **c** A western blotting image and the quantitative analysis of HIF-1α protein expression. The data are shown as the mean ± SEM, *p < 0.05. **d** The mRNA expression of VEGF-A under high glucose conditions for 3 days. The data are shown as the fold change compared to the 25 mM-treated control group and are expressed as the mean ± SEM, *p < 0.05. **e** VEGF secretion under high glucose conditions for 3 days, as determined by ELISA. The data are shown as the fold change compared to the 25 mM-treated control group and are expressed as the mean ± SEM, *p < 0.05. **f** The mRNA expression of the VEGF receptor VEGFR1 under high glucose conditions for 3 days. The data are shown as the mean ± SEM, *p < 0.05. **g** The mRNA expression of the VEGF receptor VEGFR2 under high glucose conditions for 3 days. The data are shown as the mean ± SEM, *p < 0.05

We observed the expression of HIF-1α through immunofluorescence staining, qPCR and western blotting after 3 days of glucose treatment. As Fig. 5a shows, high glucose treatment led to the stronger expression of HIF-1α as determined by fluorescence, and the lentiviral-induced overexpression of CGRP resulted in weaker expression compared to that in the empty vector group. Similarly, exposure to high glucose significantly increased the mRNA and protein expression of HIF-1α (*p *< 0.05), and lenti-CGRP displayed an obvious inhibitory effect on HIF-1α mRNA and protein expression under high glucose conditions (*p *< 0.05) (Fig. 5b, c). However, under normal glucose conditions, lenti-CGRP enhanced the mRNA expression of HIF-1α (*p *< 0.05) (Fig. 5b). Accordingly, high glucose was shown to increase the mRNA expression and secretion of VEGF through PCR and ELISA results (*p *< 0.05). Lenti-CGRP reduced the increases in VEGF-A mRNA expression and VEGF secretion (*p *< 0.05) (Fig. 5d, e). Interestingly, consistent with HIF-1α expression under normal glucose conditions, lenti-CGRP also increased the mRNA expression and protein secretion of VEGF (*p *< 0.05) (Fig. 4d, e). We also tested the mRNA expression of the main VEGF receptors vascular endothelial growth factor receptor 1 (VEGFR1) and vascular endothelial growth factor receptor 2 (VEGFR2). Lenti-CGRP was found to have an effect on VEGFR2 but not VEGFR1 by reducing its mRNA expression level under high glucose conditions (*p *< 0.05) (Fig. 5f, g).

### Lenti-CGRP partly blocked the high-glucose-induced phosphorylation of ERK1/2 and p65

**Fig. 6:**
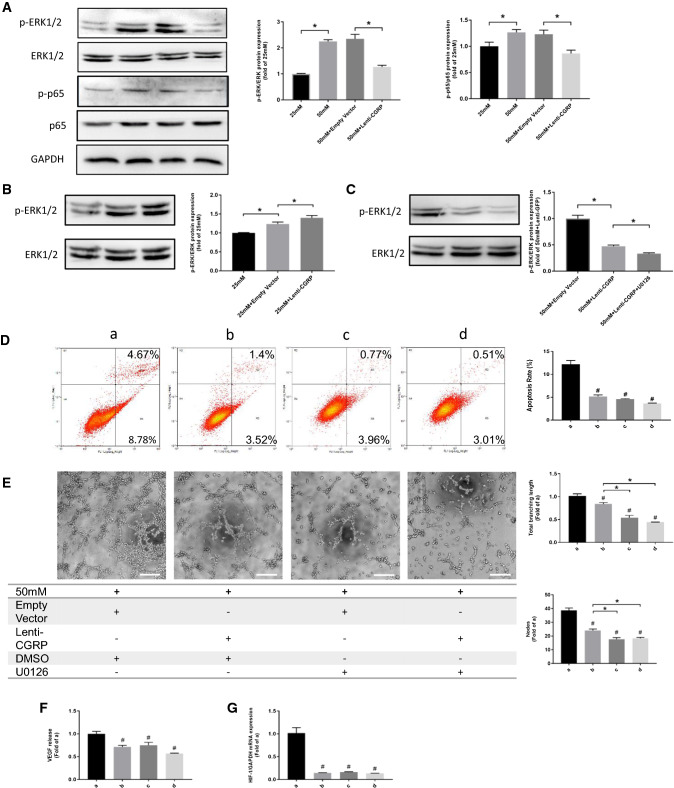
Lenti-CGRP suppressed the high glucose-induced phosphorylation of ERK1/2 and p65. **a** Representative western blotting images of p-ERK1/2, ERK1/2, p-p65, p65 and GAPDH after 3 day glucose treatment. The data are shown as the mean ± SEM, *p < 0.05. **b** Western blotting images of p-ERK1/2 and ERK1/2 under 25 mM glucose conditions for 3 days. **c** Typical western blotting images of p-ERK1/2 and ERK1/2 in the lenti-CGRP group after treatment with the ERK1/2 inhibitor U0126 for 30 min under 50 mM glucose conditions for 3 days. The data are shown as the mean ± SEM, ^#^*p* < 0.05. **d** The results of the annexin V/PI cell apoptosis assay under 50 mM glucose conditions for 5 days. The data are shown as the mean ± SEM. ^#^*p* < 0.05 vs the 50 mM glucose-treated control group. **e** Representative images of the Matrigel assay upon U0126 treatment under 50 mM glucose conditions. The data are shown as the mean ± SEM. ^#^p < 0.05 vs the 50 mM glucose-treated control group, *p < 0.05. Scale bar = 200 µm. **f** VEGF secretion under high glucose conditions upon U0126 treatment for 3 days, as determined by ELISA. The data are shown as the mean ± SEM. ^#^*p* < 0.05 vs the 50 mM glucose-treated control group. **g** The mRNA expression of HIF-1α under high glucose conditions upon U0126 treatment for 3 days. The data are shown as the mean ± SEM, ^#^*p* < 0.05 vs the 50 mM glucose-treated control group

The western blotting results indicated the upregulation of ERK1/2 phosphorylation under high glucose conditions (50 mM) compared to 25 mM glucose conditions (*p *< 0.05). Lenti-CGRP downregulated the phosphorylation of ERK1/2 after 50 mM glucose treatment (*p *< 0.05). Similarly, the increased phosphorylation of the downstream factor p65 was observed in the 50 mM glucose-treated group (*p *< 0.05). Phosphorylation was reduced by lenti-CGRP (*p *< 0.05) (Fig. 6a). However, under normal glucose conditions, lenti-CGRP induced the phosphorylation of ERK1/2 (*p *< 0.05) (Fig. 6b). To investigate the extent of the inhibitory effect of lenti-CGRP, we added the ERK1/2 inhibitor U0126 to the lenti-CGRP-treated group. On the basis of lenti-CGRP treatment, the western blotting results indicated a continuous downregulation of the phosphorylation of ERK1/2 (*p *< 0.05) (Fig. 6c). Furthermore, while lenti-CGRP displayed an inhibitory effect on cell apoptosis and tube formation (*p *< 0.05), the inhibition of ERK1/2 by the inhibitor U0126 also led to lower apoptosis levels and less tube formation (*p *< 0.05) under high glucose conditions. Neither lenti-CGRP + inhibitor U0126 group nor U0126 group showed stronger effects on cell apoptosis, which indicated that lenti-CGRP had a strong inhibitory impact similar to that of U0126 (*p *> 0.05) (Fig. 6d). However, regarding tube formation, U0126 displayed a stronger effect than lenti-CGRP group, and compared to lenti-CGRP group, lenti-CGRP + U0126 further decreased the branching length and number of nodes (*p *< 0.05), indicating the partial inhibitory role of lenti-CGRP (Fig. 6e). The ELISA results of VEGF secretion and the PCR result of HIF-1α expression showed that the inhibition of lenti-CGRP had similar inhibitory effect compared to that of U0126 (Fig. 6f, g).

## Discussion

Diabetes mellitus is a chronic metabolic disease characterized by chronic exposure to hyperglycemia, and the morbidity and mortality of diabetes largely lie in its vascular complications [27]. Both type 1 diabetes and type 2 diabetes are believed to have an impact on brain structure and function [28]. Hyperglycemia-triggered cerebral microangiopathy is believed to contribute largely to the increasing risk of diabetic neurologic disorders such as cognitive impairment [29, 30]. It has been reported that cerebral microangiopathy may begin with changes in blood dynamics, followed by impaired vasodilation, increased expression of adhesion and inflammatory cytokines, elevated adhesion, and even atherosclerosis. Basically, the cerebral microvascular endothelial cells undergo a series of metabolic changes under hyperglycemia. However, augmented cerebral neovascularization may be different from the neovascularization of other vascular beds under diabetic conditions because it has its own neurovascular unit [2]. Few studies on cerebral vascularization under high glucose conditions have been carried out until recently. Nevertheless, changes in brain microvascular endothelial cells and the underlying mechanism under high glucose conditions remain to be well understood.

Calcitonin gene-related peptide, a neuropeptide secreted from the end of a sensory nerve, plays an important role in vascular tone. Previous studies have reported its role in the proliferation [20, 21, 31], angiogenesis [20, 21], and vasodilation [31, 32] of vascular cells. However, there have been no current studies on its possible role in microvascular endothelial cells under high glucose conditions. Previous studies have reported decreased expression of CGRP under diabetic conditions [24]. In this study, we observed a relatively weaker expression of the main CGRP receptor Ramp1 by cell immunofluorescence and qPCR. Considering that Ramp1 expression is decreased under high glucose conditions, we overexpressed the CGRP gene in b.END3 cells by lentiviral transduction to explore its possible role in high-glucose-induced damage.

Previous studies have shown that CGRP improves the proliferation of HUVECs [21, 33] in vitro and rat endothelial cells in vivo [31]. Through the CCK-8 assay, lenti-CGRP had no obvious effect on cell viability at any time point under either normal or high-glucose conditions. In addition to confirming the successful transduction of the lentivirus, we insured that the transduction of the empty vector and lenti-CGRP vectors used in this study did not harm the viability of the b.END3 cells. Moreover, similar to other diabetic vascular changes, diabetes leads to vascular regression in the late stage when apoptosis of endothelial cells plays an essential role. In this study, apoptosis of b.END3 cells was measured by the annexin/PI method. Calcitonin gene-related peptide was reported to only regulate cardiomyocyte survival in past studies [23], and in this study, CGRP was mainly observed to have a protective effect against endothelial cell apoptosis under high glucose conditions for 5 days.

The angiogenic capability of cerebral microvascular endothelial cells under high glucose conditions in vitro is rarely discussed. Many in vivo studies have demonstrated increased yet dysfunctional cerebral neovascularization in diabetes [34–36]. Prakash et al. isolated brain microvascular endothelial cells from diabetic rats and interestingly discovered an increase in angiogenesis and migration, which is in accordance with the in vivo study [35]. However, until now, little has been elucidated regarding the cerebral microvascular endothelial cells and their biological behavior under high glucose conditions, as many past studies have paid more attention to the mechanism of the angiogenesis of retinal or other coronary endothelial cells. In our study, we found that high glucose mainly affected tube formation in b.END3 cells in vitro by affecting both node formation and total branching length. Calcitonin gene-related peptide was found to stimulate angiogenesis under normal glucose conditions mainly by affecting the total branching length, which is consistent with the findings of past studies [20]. However, CGRP reduced the number of nodes and branches under high glucose conditions, whereas it tended to form, on average, longer branches. Similarly, lenti-CGRP did not accelerate cell migration under high glucose conditions, while CGRP significantly stimulated migration under normal glucose conditions.

Oxidative stress induced by hyperglycemia has been reported to play a key role in diabetic vascular complications. On the one hand, the production of reactive oxygen species is enhanced, and on the other hand, the antioxidant defense system is decreased [3]. It has been suggested that CGRP can attenuate the high-glucose-induced ROS level of Schwann cell lines treated with high glucose, indicating a possible role of CGRP in diabetic neuropathy [25]. Similarly, we also found that CGRP partly rectified the increase in ROS production in b.END3 cells under high glucose conditions. High glucose induces the production of advanced glycation end products (AGEs), which combine with RAGE on the cell membrane, playing a crucial role in increasing oxidative stress in diabetic endothelial dysfunction by decreasing MnSOD and eNOS activity [8]. Nitric oxide activity is not only an oxidative signal but also a signal for vasodilation in the endothelium. Calcitonin gene-related peptide has been reported to exert its vasodilator role through iNOS activity [16]. Therefore, we also investigated the mRNA expression of iNOS and eNOS in response to high glucose. We found that lenti-CGRP could antagonize the high glucose-induced increase in iNOS levels and the decrease in eNOS levels. Additionally, regarding NADPH oxidase, the increased O_2_- production leads to the decreased bioavailability of SOD [37]. In addition, the overexpression of MnSOD has been reported to provide protection against diabetes-induced oxidative stress in retinal endothelial cells [38]. Consistent with previous studies, high glucose reduced the relative MnSOD protein level in b.END3 cells and this reduction was improved by lenti-CGRP to a certain extent. All these results indicated the potentially protective role of lenti-CGRP against high-glucose-stimulated oxidative changes in b.END3 cells.

In the diabetic brain, the decreased blood flow and severe oxidative stress create a relatively hypoxic microenvironment with a quick upregulation of HIF-1 [2]. The augmentation of HIF-1 may be an initial reaction to the onset of diabetes mellitus. In series, hyperglycemia induces the upregulation of VEGFA and increases capillary density. Yan et al. investigated the role of HIF-1 in b.END3 cells exposed to high glucose and came to the conclusion that the upregulation of HIF-1, as an upstream gene of VEGF, plays an important role in high-glucose-induced blood–brain barrier (BBB) damage in relation to tight junction proteins [13]. We also investigated whether CGRP could modulate HIF-1α expression. Through the results of immunofluorescence staining, PCR and western blotting, we found that HIF-1α mRNA and protein expression levels were indeed comparatively high, and CGRP downregulated the expression of HIF-1α early after exposure to high glucose. In addition, we also observed an increase in VEGF release, which was associated with increased angiogenesis, after 3 days of high glucose exposure. The oversecretion of VEGF was partially blocked by lenti-CGRP. Afterwards, we investigated the mRNA expression of the main VEGF receptors, VEGFR1 and VEGFR2, to determine whether CGRP has a possible influence on VEGF receptors. Early after exposure to high glucose, the mRNA expression of both receptors was enhanced. Lenti-CGRP mainly affected VEGFR2, but it did not have much of an effect on VEGFR1 expression. The findings indicated that CGRP worked mainly through VEGFR2 to regulate VEGF release. Interestingly, we found that under normal glucose conditions, CGRP enhanced the mRNA expression of HIF-1α and VEGF mRNA expression and secretion, which might indicate that the pro-angiogenic effect of CGRP under normal glucose conditions may be related to its regulation of HIF-1α/VEGF. These results partially agree with those of past studies regarding the angiogenic effect of CGRP on endothelial cells [21].

The ERK cascade is mainly considered to be correlated with cell proliferation and survival. Meanwhile, p65, as a downstream transcription factor that responds to oxidative stress, has been reported to be involved in inflammatory responses and apoptosis in endothelial cells. Exposure to high glucose has been reported to activate ERK1/2 as well as its downstream factor p65 in vascular cells [11, 12]. Calcitonin gene-related peptide has been reported to protect vascular smooth muscle cells and cardiomyocytes from oxidative stress through the ERK1/2 and p38 signaling pathways [22, 23]. Therefore, we speculated that CGRP might also function through ERK signaling under high glucose conditions. In the present study, lenti-CGRP blocked the enhanced phosphorylation of ERK1/2 triggered by high glucose while lenti-CGRP increased the phosphorylation of ERK1/2 under normal glucose conditions. This contrast demonstrates the difference between the regulation on HIF-1/VEGF by CGRP under various glucose conditions, which may explain the opposite angiogenic role of CGRP on tube formation. In addition, the phosphorylation of downstream p65 is changed in a similar way as ERK1/2 under high glucose conditions. We then added the ERK1/2 inhibitor U0126 to explore the extent of inhibition by lenti-CGRP. The transduction of lenti-CGRP inhibited cell apoptosis and tube formation under high glucose conditions by suppressing ERK activation. Furthermore, the addition of ERK inhibitor to the lenti-CGRP-treated group did not have a stronger effect on cell apoptosis than the inhibitor alone or lenti-CGRP. However, the stronger inhibitory effects of U0126 than lenti-CGRP on tube formation indicated that the modulation of angiogenesis by CGRP may only occur partially through ERK1/2. Additionally, the inhibition of ERK led to the decreased HIF-1α mRNA expression and subsequently decreased VEGF secretion, confirming the relationship between ERK and HIF-1α/VEGF. All of these results indicated that CGRP possibly affected apoptosis and angiogenesis of b.END3 cells under high glucose conditions through ERK/HIF-1/VEGF signaling.

Our present study has some limitations. For example, considering the decreased expression of Ramp1 in b.END3 cells under high glucose conditions, we focused only on the overexpression of CGRP in b.END3 cells. The effects of knock-down or knock-out of CGRP in b.END3 cells remain to be investigated in future studies. Furthermore, the experimental conditions we used in this study may be a little different from previous studies [13]. We used 25 mM glucose as normal glucose control and 50 mM glucose as high glucose treatment considering the b.END3 cells we used in this experiment are required to be cultured in a growth medium with 25 mM glucose. However, we set the same glucose concentration difference as 25 mM which has been reported in previous studies using 5 mM glucose as normal glucose control and 30 mM glucose as high glucose treatment [13].

In conclusion, our study demonstrated that the lentivirus-mediated overexpression of CGRP protects mouse cerebral microvascular endothelial cells from high-glucose-induced cell apoptosis, increased tube formation and related oxidative changes possibly by partly inhibiting ERK/HIF-1/VEGF signaling. Our study might help to elucidate the protective role of CGRP in diabetic cerebral microvascular cells.
